# Never again? Challenges in transforming the health workforce landscape in post-Ebola West Africa

**DOI:** 10.1186/s12960-019-0351-y

**Published:** 2019-03-07

**Authors:** Barbara McPake, Prarthna Dayal, Christopher H. Herbst

**Affiliations:** 1 Nossal Institute for Global Health, University of Melbourne, Level 5, 333 Exhibition St, Parkville, 3010 Australia; 2 The World Bank, 1818 H Street, NW, Washington, DC, 20433 United States of America

**Keywords:** Human resources for health, Health workforce, Health system, Ebola, West Africa

## Abstract

**Background:**

The 2013–2014 West African Ebola outbreak highlighted how the world’s weakest health systems threaten global health security and heralded huge support for their recovery. All three Ebola-affected countries had large shortfalls and maldistribution in their health workforce before the crisis, which were made worse by the epidemic. This paper analyzes the investment plans in Liberia, Sierra Leone, and Guinea to strengthen their health workforces and assesses their potential contribution to the re-establishment and strengthening of their health systems. The analysis calculates the plans’ costs and compares those to likely fiscal space, to assess feasibility.

**Methods:**

Public sector payroll data from 2015 from each country was used for the workforce analysis and does not include the private sector. Data were coded into the major cadres defined by the International Standard Classification of Occupations (ISCO-88). We estimated health worker training numbers and costs to meet international health worker density targets in the future and used sensitivity analysis to model hypothetical alternate estimates of attrition, drop-outs, and employment rates.

**Results:**

Health worker-to-population density targets per 1000 population for doctors, nurses, and midwives are only specified in Liberia (1.12) and Guinea’s (0.78) investment plans and fall far short of the regional average for Africa (1.33) or international benchmarks of 2.5 per 1000 population and 4.45 for universal health coverage. Even these modest targets translate into substantial scaling-up requirements with Liberia having to almost double, Guinea quadruple, and Sierra Leone having to increase its workforce by seven to tenfold to achieve Liberia and Guinea’s targets. Costs per capita to meet the 2.5 per 1000 population density targets with 5% attrition, 10% drop-out, and 75% employment rate range from US$4.2 in Guinea to US$7.9 in Liberia in 2029, with projected fiscal space being adequate to accommodate the proposed scaling-up targets in both countries.

**Conclusions:**

Achieving even a modest scale-up of health workforce will require a steady growth in health budgets, a long-term horizon and substantial scale-up of current training institution capacity. Increasing value-for-money in health workforce investments will require more efficient geographical distribution of the health workforce and more consideration to the mix of cadres to be scaled-up.

**Electronic supplementary material:**

The online version of this article (10.1186/s12960-019-0351-y) contains supplementary material, which is available to authorized users.

## Background

As the 2014 West African Ebola outbreak was finally declared over, a period of unprecedented attention to global health security began, with four global commissions in a space of less than 1 year [1]. Prominent among the conclusions of those commissions was the need to ensure that countries meet, and are supported to meet, their obligations under the International Health Regulations to fund and build core health system capacities [1]. Kluge et al. summarizes the emerging consensus concerning the inter-dependence of health systems and health security: Universal Health Coverage (UHC)—or the achievement of an effective health system—supports health security by preventing outbreaks of disease for example through effective case management by a sufficient and well-functioning health care workforce, while health security supports UHC by avoiding crises that undermine health system functioning [2].

Widespread consensus in the immediate aftermath suggested a watershed moment had arrived whereby the importance of the world’s weakest health systems for global health security was understood and from where a sea change in support for the recovery of those health systems would emerge. For example, Andrew Green, reporting for the Lancet in 2016 suggested “Now, with the support of an international community awakened to the global security threat, there is the chance to begin filling … gaps. Each (of the three West African countries) has introduced strategic plans calling for not just health system fixes, but improvements to all of the conditions that facilitated Ebola’s explosion” [3].

Key to any analysis of pre-Ebola health system weakness and post-Ebola priority targets for health system investment are shortfalls in the health workforce in all three countries. Long before the outbreak struck, there were extensive analyses of the nature of those shortfalls in each country and proposals for resolving those. Liberia had made the most significant progress prior to the outbreak. An Emergency Human Resources Plan (EHRP) had been implemented between 2007 and 2011, significantly increasing the numbers of nursing and midwifery cadres in particular. However, in 2010, the number of clinical health workers per 1000 population had only reached 1.3, far short of any international benchmarks. Less progress was made under the EHRP in relation to the geographical distribution of staff [4]. Sierra Leone’s health workforce had fallen to a level per 1000 population of 0.2 by 2008, and this workforce was heavily concentrated in urban areas [5]. Jansen et al. showed that Guinea had less than half the health staff needed at the beginning of 2014 while projections contrasted growing need with declining workforce numbers, a particularly acute divergence for nurses and midwives. Rural areas projected much larger shortfalls than urban while Conakry was projected to contain a large surplus of health workers [6].

Given such small numbers in the health workforce in all three countries: Liberia for example had 4653 clinical health workers in 2010, Sierra Leone had 2672 in 2008, and Guinea approximately 3000 in 2014 [4–6]; deaths of an estimated 418 health workers during the Ebola crisis across all three countries are significant [7]. By anecdote at least, these losses were compounded by health workers abandoning their posts during the crisis,^1^ although other research attests to the commitment and resilience of health staff in the face of the enormous challenges posed by the outbreak, including pressure from families and communities to abandon their posts [8–10]. Quantitative data related to the workforce losses other than through mortality are largely absent.

In the light of all these factors, this paper aims to document and assess the plans in each of the three countries to strengthen the health workforce situation as a component of the re-establishment and strengthening of the health systems that promised to follow the crisis.

As the Ebola virus disease started to recede, post-Ebola health system investment plans were developed through extensive consultations in all three countries. Prepared with strong government leadership, key stakeholders, and support from the international community, these plans outline the most critical investments needed to strengthen the health system in each country [11]. Among other things, these plans seek to expand the availability of well-performing health workers at all levels of the health system and contain workforce scaling-up targets for each country to aim to achieve.

These plans are dissected in this paper from the perspective of their potential contribution to supporting a revitalized health system, including the extent to which they will contribute to the resolution of the pronounced distributional problems described above. The paper further costs and compares the plans to likely fiscal space (the ability of the government to increase spending on health) to assess their feasibility.

## Methods

All three countries analyzed in this paper are low-income economies as classified by the World Bank with relatively high infant and under-five mortality rates (Table 1).

**Table 1:** Basic demographic, economic and health indicators comparing the three countries

	Guinea	Liberia	Sierra Leone
Total population (thousands)	12 717	4732	7557
GDP per capita 2017 (US$)	824	545	463
GDP growth rates (annual %)	10.6	2.5	4.2
Infant mortality rate (per 1000 live births)	66	59	94
Under-five mortality rate (per 1000 live births)	102	80	134

Source: compiled by authors from the World Bank Indicators 2017 https://databank.worldbank.org/data/reports.aspx?source=2&series=NY.GDP.MKTP.CD&country=#

*Health workers* in this analysis are defined as all cadres employed in the service of health. This includes care providers (such as doctors, nurses, and midwives) as well as allied health professionals and administrative and support staff. The central analysis of this report, however, focuses on doctors, nurses, and midwives because of the existing focus in the international literature on densities of these cadres and the evidence of their relevance to health service delivery outcomes (for example, [12, 13]).

Data were coded into the major cadres defined by the International Standard Classification of Occupations (ISCO-88). Public sector payroll data from 2015 were used for the health workforce analysis. Data were obtained by the World Bank and WHO from the relevant government departments in the three countries [11]. Although data were verified by government stakeholders, no formal quality assessment was undertaken. Payroll data are generally reflective of the public health workforce on government payroll; this is comparable across the three countries and likely represents the majority of the workforce in the country. However, there are shortcomings. Payroll data include some “ghost workers” (those workers listed on the payroll but who are not actually working in the system), and they exclude health workers who exclusively work in the private sector (largely concentrated in the capitals in all three countries) or who work in the public sector but are paid by NGOs or provide services voluntarily. The 2014 census data in Guinea, for example, lists 4566 health workers in the public sector paid by NGOs. In Sierra Leone, this estimate is 9000 health workers [11]. However, these are mostly community health workers (CHW) and therefore have little bearing on the analysis of doctors, nurses, and midwives. Payroll data from 2015 do not reflect the results of recent payroll audits that have been completed in Liberia and Sierra Leone, in part to weed out “ghost workers.” Dual practice is likely to be common so that many private sector workers are captured on the government payroll. This is one respect in which the data required for a complete labor market analysis were incomplete or missing. The assumptions and scenarios developed in relation to health worker training numbers were also all based on missing or imperfect information. We managed the lack of reliable information on attrition by using sensitivity analysis to model hypothetical alternative estimates of attrition, drop-outs, and employment rates for illustrative purposes. Lack of defined and cross-country comparability of rural and urban areas in Sierra Leone and Liberia were managed by defining the region including the capital city as urban and all other areas as rural. This resulted in urban populations of 34.8% in Guinea, 32.2% in Liberia and 18.9% in Sierra Leone. Annual salary estimates were also obtained from payroll data from government health departments in Liberia, Sierra Leone, and Guinea. Training cost estimates were sourced from the One Health Tool used by the governments in Guinea and Liberia for developing their investment case plans [11]. Training cost data for Sierra Leone were unavailable and regional costs used in their place. The analysis and figures should thus be interpreted with these limitations in mind.

## Results

### Health workforce stock and distribution: the current public sector situation

**Fig. 1:**
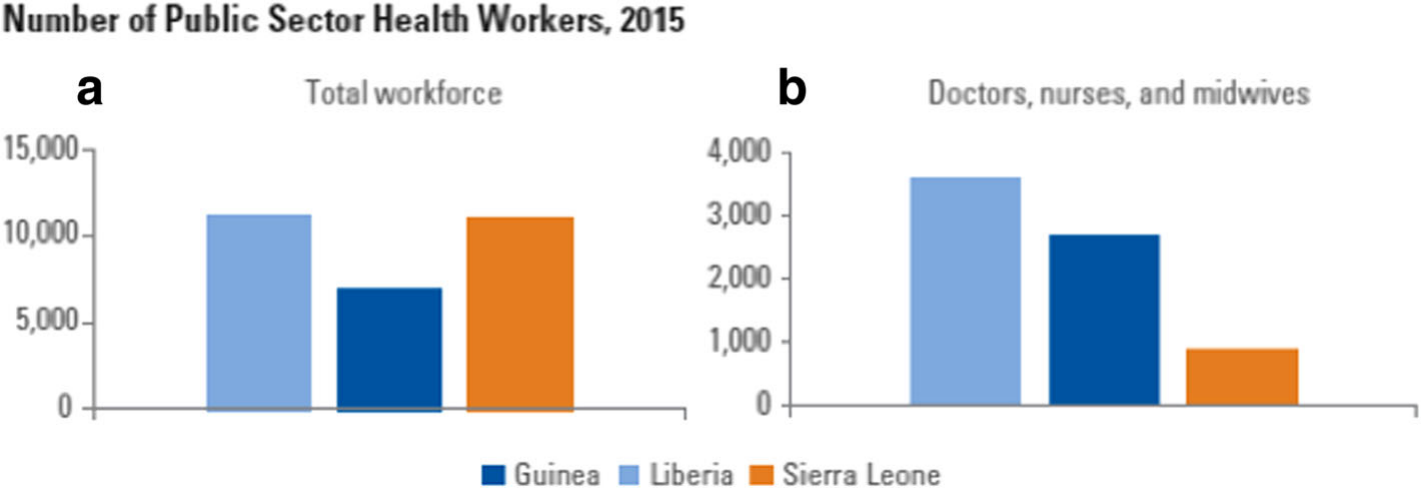
Number of public sector health workers, 2015

As indicated by analyses from before the outbreak, the stock of health workers in all three countries is extremely low, though Liberia fares comparatively better on this front than Sierra Leone or Guinea in terms of raw numbers of doctors, nurses, and midwives only (Fig. 1). Although Guinea has the smallest stock of combined health workers (when all health worker categories are included), it has a large stock of community health volunteers and the largest stock of doctors of the three countries. Liberia has the largest stock of mid-level cadres, and Sierra Leone has the largest stock of low-level cadres.

These differences are exacerbated when population levels and the ratio of health workers to population are taken into account (Additional file 1): density levels of doctors, nurses, and midwives per 1000 population in Liberia are higher than they are in the other two. The extremely low level of health workers in all three countries is evident from comparison with regional averages (Table 2). Liberia, with the largest density of all three countries, is only close to half the African average (with 0.77), with Guinea and Sierra Leone falling even further behind (with 0.20 and 0.15, respectively).

**Table 2:** Average densities of doctors, nurses, and midwives, per 1000 population, 2013

WHO region/country	Physicians	Nurses and midwives	Total
Africa	0.24	1.09	1.33
Americas	2.29	5.49	7.78
Eastern Mediterranean	1.01	1.42	2.43
Europe	3.25	6.81	10.06
South Asia	0.58	1.24	1.81
East Asia	1.87	2.51	4.37
Western Pacific	1.40	2.08	3.48
Global	1.36	2.75	4.11
Liberia	0.03	0.74	0.77
Guinea	0.10	0.1	0.20
Sierra Leone	0.04	0.11	0.15

Source: For WHO Regions: Global Health Observatory, World Health Organization (http://apps.who.int/ghodata/#); For Liberia, Guinea, and Sierra Leone—authors calculations based on the current stock available from public payroll and 2015 population estimates (CIA database)

**Fig. 2:**
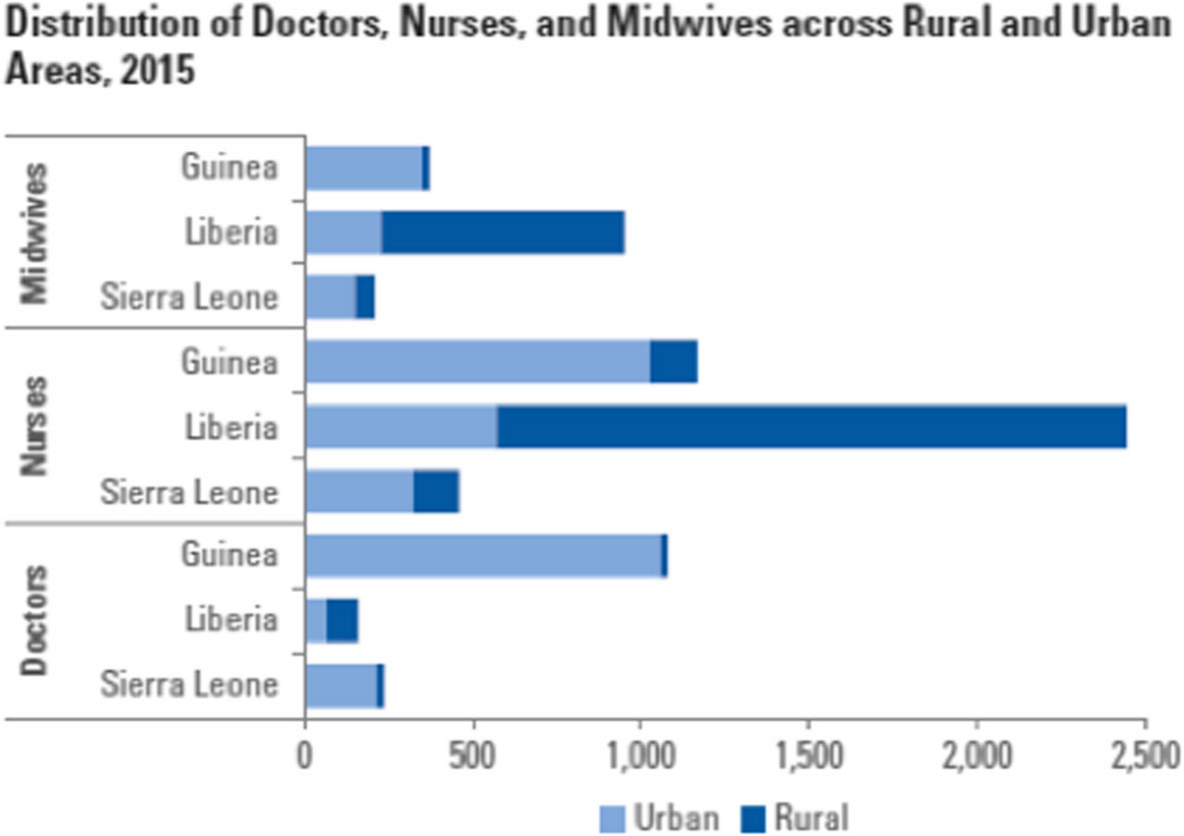
Distribution of doctors, nurses, and midwives across rural and urban areas, 2015

The distribution of health workers is uneven in all three countries, although Liberia’s workforce is more evenly distributed than the others, with 57% of doctors in rural areas, and 43% in urban areas (the population distribution is 68% rural and 32% urban). In contrast, in Guinea 98% of doctors and 88% of nurses reside in urban areas, where only 36% of the population live; and in Sierra Leone, 92% of doctors and 72% of nurses reside in urban areas, where only 18% of the population live (Fig. 2).

### Health worker scaling-up ambitions and implications by investment plans

Health worker scaling-up plans in Guinea and Sierra Leone run until 2024 and 2025, respectively; in Liberia, the plan extends until 2021. We assessed the implications of the density targets identified in the investment plans in relation to population threshold densities associated with increased service delivery coverage, graduate production, and cost.

The investment plans of Guinea and Liberia mention specific health worker-to-population density targets to be achieved; this target is missing in Sierra Leone’s investment plan. Liberia’s stated target is 1.4 doctors, nurses, midwives, and physician assistants per 1000 population. Removing physician assistants from this scenario produces a target density of 1.12 per 1000 for doctors, nurses, and midwives alone. Guinea’s stated target is 0.26 doctors per 1000 population, 0.26 nurses per 1000 population, and 0.26 midwives per 1 000 population—this produces a target density of 0.78 per 1000 population for doctors, nurses, and midwives by 2024. Given the absence of a stated target in Sierra Leone, the implications of using the densities proposed by the other two countries are used as a proxy in further analyses.

**Fig. 3:**
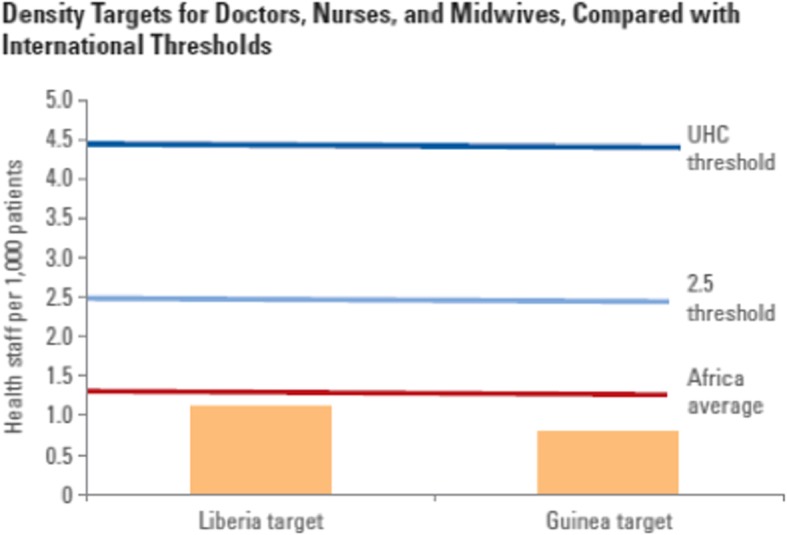
Density targets for doctors, nurses, and midwives, compared with international thresholds

The density threshold targets set in the investment plans are far below both the current regional average and international thresholds associated with improved health outcomes and service delivery indicators (Fig. 3). Commonly used international density thresholds focus on doctors, nurses, and midwives. All of the targets are substantially lower than the current regional density average of 1.33 doctors, nurses, and midwives per 1000 population. They are also significantly lower than a commonly used workforce density threshold level of 2.5 per 1000 population [12], which is associated with improved service delivery coverage, as well as a new threshold of 4.45 per 1000 population which has been proposed in association with universal health coverage [14]. Hence, targets do not meet the minimum levels required to achieve adequate service delivery across the population.

Nevertheless, even the modest density targets in each investment plan translate into substantial scaling-up requirements for health workers, particularly in Guinea and Sierra Leone (Table 3). To achieve the density targets identified in the investment plans, Liberia would have to close to double its number of doctors, nurses, and midwives; annual growth rates for each of the three cadres would have to be 9.6% to reach the proposed density targets. Guinea would have to more than quadruple its health workforce; Guinea’s annual growth rates would have to be 17% for each cadre. If Sierra Leone were to aim to meet the same targets as Guinea and Liberia, it would have to increase its current stock by sevenfold (to meet Guinea’s density threshold) and more than tenfold (to meet Liberia’s): its annual growth rates would have to be 21.5% or 26%, depending on the density target chosen. It should be noted, however, that these growth rates are premised on small initial numbers.

**Table 3:** Investment plan density target implications

Country	Implications	Doctors	Nurses	Midwives	Total
Liberia	Current stock (2015)	158	2445	952	3555
	Total stock needed to reach target density (1.12 per 1000) in 2021 (% annual growth)	274 (9.7%)	4245 (9.7%)	1653 (9.7%)	6172
Guinea	Current stock (2015)	1111	1168	372	2651
	Total stock needed to reach target density (0.78 per 1000) in 2024 (% annual growth)	4567 (17%)	4801 (17%)	1529 (17%)	10 897
Sierra Leone	Current stock (2015)	234	450	208	892
	Required for target density (0.78 per 1000) in 2025 (% annual growth)	1638 (21.5%)	3151 (21.5%)	1456 (21.5%)	6245
	Required for target density (1.12 per 1000) by 2025 (% annual growth)	2352 (26%)	4524 (26%)	2091 (26%)	8967

Note: In each case, it is assumed that the current staff ratios across the three cadres will not change, so the growth rates for each cadre are the same. Note that Liberia’s investment plan target has been adjusted from 1.4 per 1000 to 1.12 per 1000 with the removal of physician assistants for the purpose of this analysis

The total and annual cost implications of the three countries pursuing their scaling-up ambitions, draw on a number of assumptions: the total cost includes both salary and training costs, and the average salary reflected on the payroll was used (Additional file 2). Where training cost was not known, the training cost for a staff group with similar earnings was used.

We modeled a baseline scenario to address attrition from the workforce, drop out from pre-service training, and uptake of employment in the public health sector at the conclusion of pre-service training. This scenario assumed 10% workforce attrition, a 20% dropout rate from training, and a 50% employment rate in the public sector. We calculated the cost of achieving the proposed targets per head of population in each country for this scenario. This was highest in Sierra Leone, followed by Liberia; and substantially lower in Guinea. In Sierra Leone, achieving a target similar to Guinea’s in 2024 would cost US$18.25 per capita annually; achieving a target similar to Liberia’s in 2024 would cost US$24.10 per capita annually. In Liberia, achieving the proposed target of 1.12 nurses, midwives, and physicians per 1000 population in 2021 would cost US$8.19 per capita annually. In Guinea, achieving the proposed target of 0.78 nurses, midwives, and physicians per 1000 population in 2024 would cost US$1.51 per capita annually.

### Comparing investment plan targets with globally set targets for scaling-up workforce

The previous section has shown the investment plan density targets are far from global targets based on estimates of requirements to achieve minimum service delivery coverage and health outcome standards. This section assesses the growth rates required and costs associated with reaching the threshold of 2.5 doctors, nurses, and midwives per 1000 population which is the more modest international target of those proposed. Because no reliable cost data were available for Sierra Leone, the cost projections were done for Guinea and Liberia only.

Table 4 shows the current numbers of doctors, nurses, and midwives in each country and how many will be required to achieve 2.5 per 1000 population density by the years 2020, 2025, and 2030, on the basis of the maintenance of the current ratios of staff across the three cadres. These numbers show that a 2020 target for achieving international targets of health workforce ratios is clearly not feasible. Setting a later target date of 2030 requires only slightly higher rates of growth than those required by the investment plans, and the following discussion is based on that target date.

**Table 4:** Numbers of workers required to meet 2.5 per 1000 population density by 2020, 2025, and 2030

Stock	Liberia			Guinea			Sierra Leone		
	Doctors	Nurses	Midwives	Doctors	Nurses	Midwives	Doctors	Nurses	Midwives
Current stock	158	2445	952	1111	1168	372	234	450	208
Total required in 2020	593	9184	3576	13108	13781	4389	4754	9142	4225
Total required in 2025	699	10 814	4211	15 049	15 821	5039	5251	10 098	4668
Total required in 2030	824	12 748	4964	17 312	18 200	5797	5804	11 162	5159

**Fig. 4:**
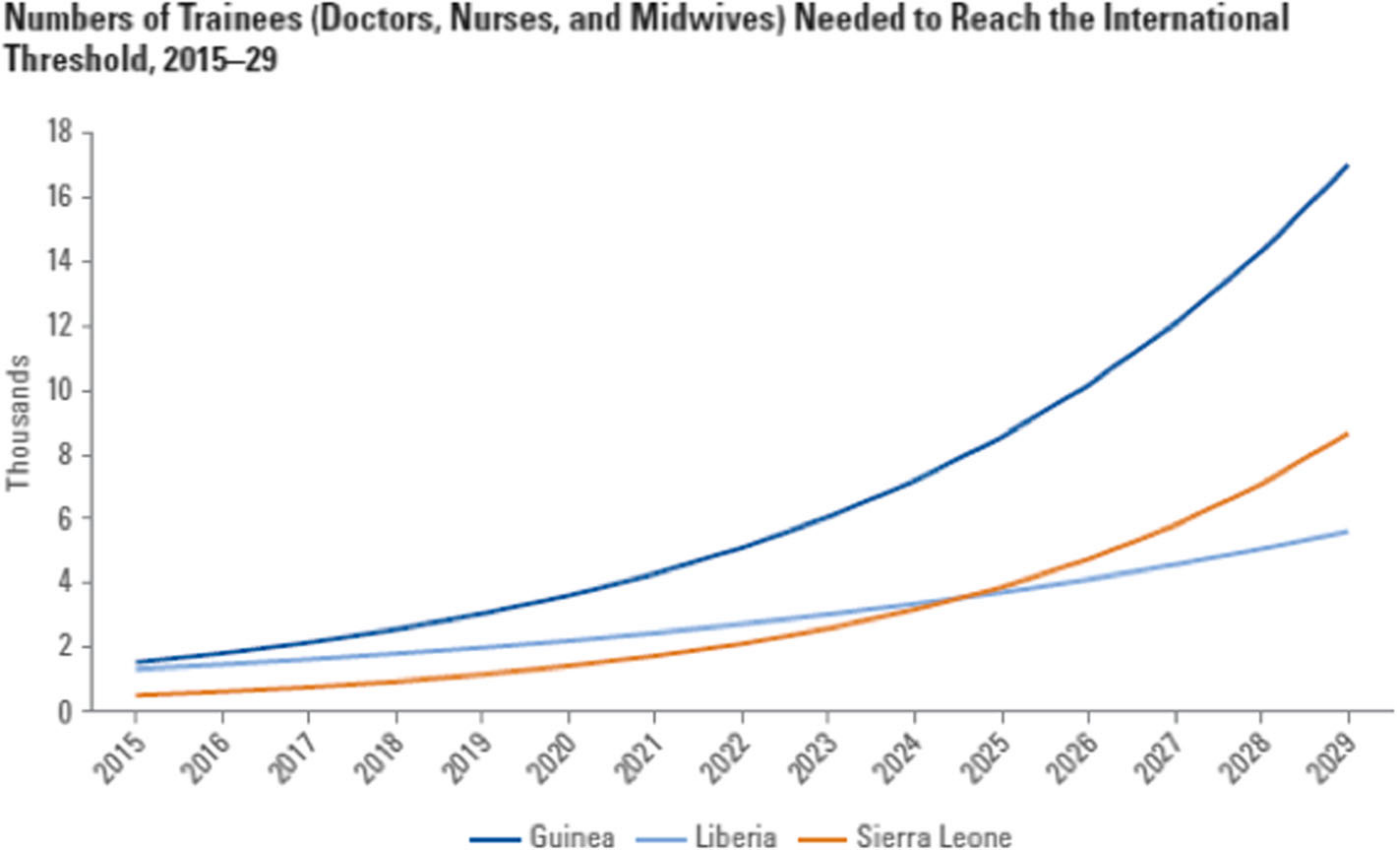
Numbers of trainees (doctors, nurses, and midwives) needed to reach the international threshold, 2015–2029

Figure 4 shows the estimated number of graduates required to meet the international threshold under the baseline attrition, training dropout, and public sector employment scenario.

Costs of achieving the 2.5 density benchmark by 2030 for Guinea and Liberia were calculated on the basis of the same assumptions as in the previous section. For this analysis, we modeled an alternative attrition, dropout, and employment scenario to the baseline scenario, halving losses at each stage, as per Table 5. Table 6 shows that the cost ranges from US$4.2 per capita in Guinea to US$ 7.9 per capita in Liberia, for the alternative scenario in 2029 (the last year in which trainees graduate to achieve the 2030 target). The differences across countries reflect large differences in cost estimates for wages and for training which are more important in the overall cost projection than the scenario differences.

**Table 5:** Hypothetical scenarios of attrition and employment percent

Scenario	Workforce attrition	Drop out of training	Employment rate
Baseline scenario	10	20	50
Alternate scenario	5	10	75

**Table 6:** Cost of achieving minimum densities of doctors, nurses, and midwives, 2015–2029 under two scenarios US dollars, millions/cost per capita

Country	Scenario	2015	2020	2025	2029
Liberia	Baseline scenario	16.2/3.6	27.2/5.1	45.6/7.3	68.9/9.6
	Alternate scenario	13.3/2.9	22.3/4.2	37.4/5.9	56.5/7.9
Guinea	Baseline scenario	6.7/0.6	15.8/1.3	37.4/2.6	74.3/4.6
	Alternate scenario	6.1/0.6	14.3/1.1	33.7/2.3	67.0/4.2

### Comparing cost estimates with fiscal space projections

This section looks at the extent to which the fiscal space for HRH (current and projected) in Guinea and Liberia is sufficient to accommodate the proposed scaling up and potential scaling up to internationally recommended density targets. Because of the lack of accurate costing data, this assessment was not done for Sierra Leone.

**Fig. 5:**
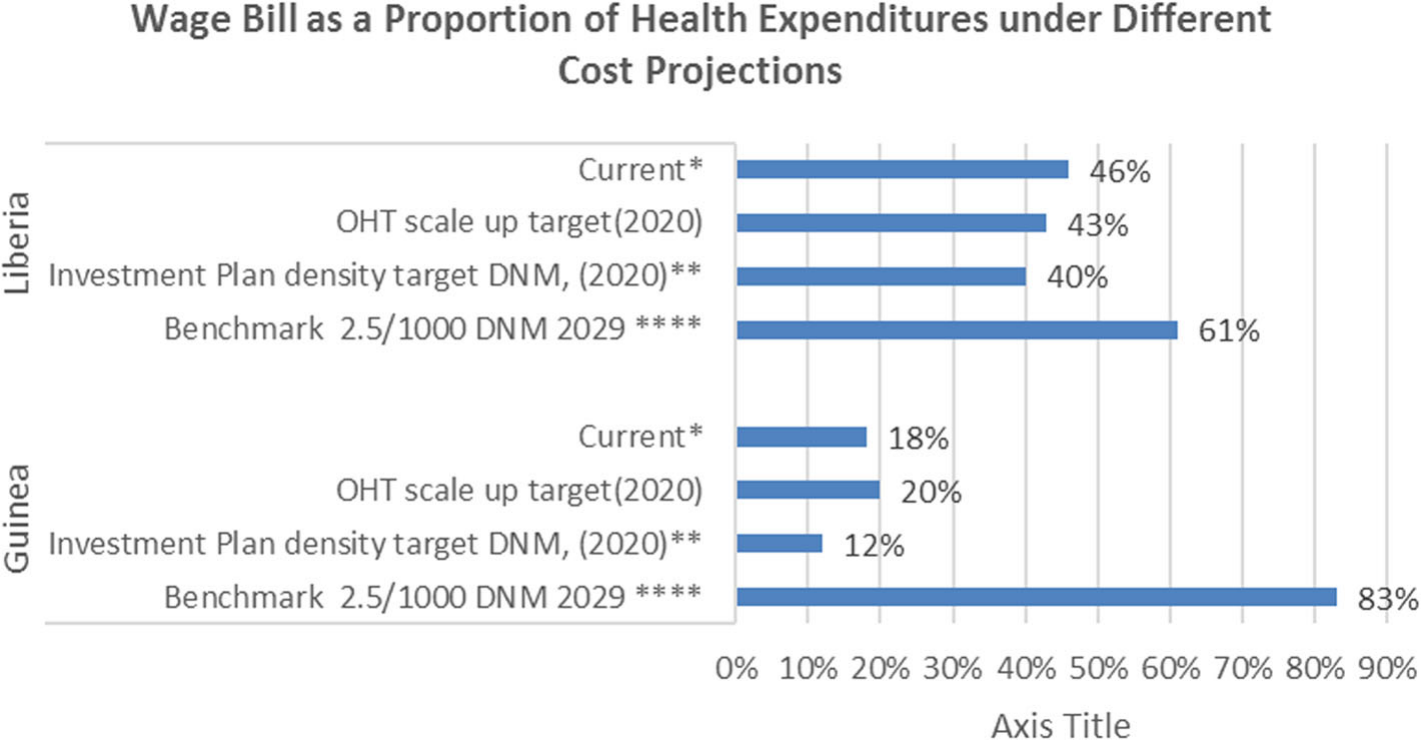
Wage bill as a proportion of health expenditures under different cost projections

GDP and government expenditures have been estimated for 2020 based on IMF projections (as of April 2016)^2^ (data provided as Additional file 3). Further projection to 2030 compares a pessimistic scenario (no growth in these indicators between 2020 and 2030) and a more optimistic scenario (5% annual growth in these indicators between 2020 and 2030) (Fig. 5). Based on the national investment plans, both Guinea and Liberia project a declining proportion of total health expenditures accounted for by the wage bill. If both countries were to achieve the desired target ratio of doctors, nurses, and midwives as outlined in their investment plans, the share of total health expenditure being absorbed by workforce costs would actually see a reduction from current levels: from 18 to 12% in Guinea and from 46 to 40% in Liberia. Overall, the projected fiscal space seems to be adequate to accommodate the proposed scaling-up targets outlined in the investment plans of both countries.

The projection of the costs associated with the ambition of achieving international doctor, nurse, and midwife density thresholds of 2.5 per 1000 population by 2030 increases the projected share of health workforce costs in total health expenditure considerably, but to shares that would not be outliers by international standards in Guinea (51%) and Liberia (38%), if growth in health budgets is projected after 2020 (see Fig. 5). Whereas this could suggest that both countries could be more ambitious in their scale-up, it is important to note that these densities reflect the levels of doctors, nurses, and midwives only and do not include all the other cadres that need to be accommodated by the public sector wage bill.

### Health workforce distribution

The Liberian investment plan includes a housing allowance for 10% of the workforce (in underserved areas) and plans to develop fair and equitable remuneration by introducing and financing a hardship allowance. The Guinean and Sierra Leonean plans discuss the aim of establishing an effective system of incentives and allocation of staff to underserved areas, but specific strategies are not defined. Both countries, however, point to the importance of carrying out labor market assessments in order to identify strategies that target solutions to address rural/urban imbalances. Moreover, both Liberia and Sierra Leone specifically emphasize the importance of developing a CHW program with the objective of ensuring greater health worker coverage in rural areas. Guinea has similar ambitions.

**Fig. 6:**
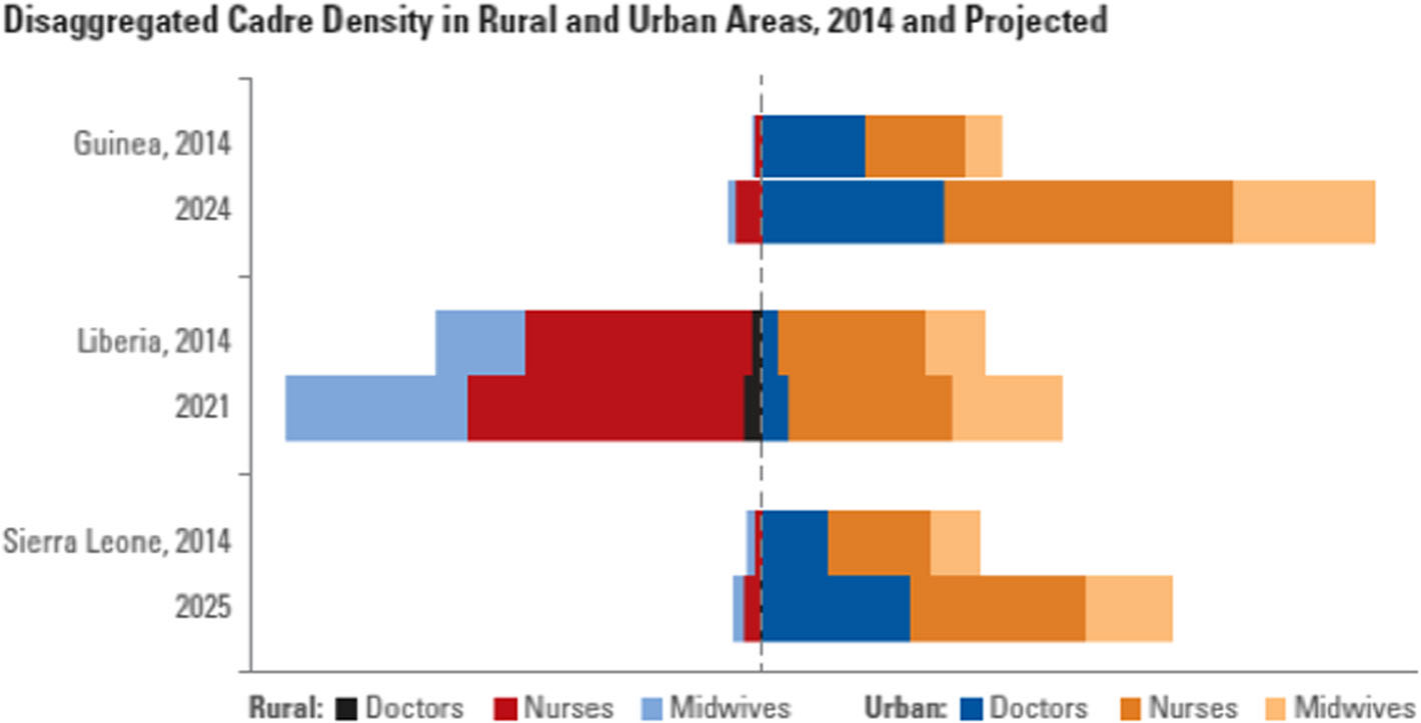
Disaggregated cadre density in rural and urban areas, 2014 and projected

Table 7 shows the workforce growth rates required for plan targets in each country, broken down by rural and urban requirements. The low base for some of the projections —for example, currently Sierra Leone has only 22 rural doctors (but 91.9% rural population)—drives the high growth rates calculated. The patterns of cadre distribution are not projected to change (see Fig. 6).

**Table 7:** Annual rural versus urban growth rates required to reach plan targets percent

Country (target year)	Doctors		Nurses		Midwives	
	Urban	Rural	Urban	Rural	Urban	Rural
Liberia (2021)	5.6	12.2	6.0	6.9	6.1	2.4
Guinea (2024)	11.3	63.0	12.3	33.7	11.5	44.3
Sierra Leone (2025)	4.2	49.9	6.8	33.8	6.8	34.3

## Discussion

There are complex challenges in achieving significant health system change in the short and medium terms. This case study shows that achieving even a modest scale-up of health workforce, a health system component on which all others rely, to the minimum levels considered to ensure delivery of even basic services, far less the level that is considered sufficient to support Universal Health Coverage requires levels of investment which will require steady growth in health budgets, a long time horizon and a substantial initial investment in scaling up training institutions. In all countries, a critical constraint is the current capacity of training institutions. All three countries currently struggle to produce even current levels of graduate output. Far more international support is needed for the three countries to address their current training capacity weaknesses, if acceptable levels of health workers are to be produced. A suitable investment on the part of “an international community awakened to the global security threat” [3] would be in supporting a significant scale-up of this capacity.

There are several caveats in relation to this conclusion. First, costs were estimated at current financial levels, and the resulting problems illustrate the importance of estimating societal (economic) costs, even if these can be less intuitive for users of the information. While Sierra Leone’s existing cost estimates were not considered reliable for inclusion, Guinea’s and Liberia’s cost levels are probably lower than an estimate of economic cost would suggest. Guinea’s estimates used, for example, were US$1200 for a doctor’s salary per year and US$2800 to train a doctor, respectively. This is probably below the rates the market would determine (the training cost also reflects low public sector salaries, the main ingredient in the structure of training school costs, for example, [15]). This allows for financial sustainability in the health budget but will produce other problems in the scale up, for example, attrition levels higher than those modeled, or motivation levels associated with poor quality of care and training. From that perspective, they are likely to be false economies, but paying at a more realistic market level might not be financially sustainable. Both Guinea and Liberia may also need to ensure that other requirements of training—related to sufficient physical, technical, and organizational capacities of health training institutions—are adequately resourced.

Interventions that focus on improving efficiency in both training institutions and the use of health staff once trained could further support a trajectory towards an adequate workforce capacity in both volume and motivation terms at a sustainable cost in all three countries. A comprehensive assessment of the potential for that is beyond the scope of the paper, but there are likely significant opportunities to make cost savings relative to the projections. Significant labor market inefficiencies: loss of trained staff to attrition; demotivated staff; high levels of absenteeism are known problems shared at least to some extent by all three countries [4, 6, 16]. Although we used the less ambitious scenario in projecting scale-up costs, real levels of attrition, drop-out, and scale-up may be higher than these, threatening the sustainability of investments. Furthermore, there are two key points within the scope of these analyses that indicate clear potential to increase the value for money in health workforce investment.

The first is the more efficient geographical distribution of health staff than any of the three countries is currently achieving. Reaching the international threshold of 2.5 doctors, nurses, and midwives per 1000 population will not produce gains in health service coverage and outcomes if those staff continue to be concentrated in urban areas to the extent implied by Fig. 2. The extremes of poor distribution exhibited by Sierra Leone and Guinea suggest not only a very significant scope to improve health outcomes by focusing on measures to improve distribution but a clear implication that the investment discussed in this paper will not be warranted at all, if these issues cannot be addressed. However, this will not reduce the costs of achieving the outcome or contribute to greater sustainability—it is best viewed as a pre-requisite for the investment to yield the intended return.

The second is the mix of cadres that it is proposed to invest in. Our projections keep the ratios of doctors, nurses and midwives constant and in line with the current ratios. Investment focused on nurses and midwives rather than on doctors will reduce costs in all countries. Scheffler et al. (2016a) estimate a need in low-income countries for a ratio of approximately 2.5:1 nurses and midwives to doctors [17]. Liberia exceeds that ratio, Sierra Leone’s ratio is similar, and Guinea is far below this ratio, suggesting scope particularly in Guinea, to reduce costs by this strategy. Investment in other cadres such as physician assistants, as is planned in Liberia, will also enable more efficient service delivery and better use of doctors, nurses, and midwives where their role is to support them. The available evidence has not quantified the extent to which investment in such mid-level cadres can substitute for doctors, nurses, and midwives, but evidence abounds that such substitution can be cost-effective [18]. A similar case can be made for CHWs and volunteers. All three countries plan substantial scale-up of these and there is evidence that such workers can be effectively used to support service delivery and health outcomes [19], but it is not clear the extent to which they can substitute for professional staff, if at all. Hence a strategy that would yield a higher rate of return or allow for a lower cost of investment to achieve the same outcomes, will involve a reassessment of appropriate ratios of doctors, nurses, and midwives and their support through investment in complementary mid-level cadres. This refocusing of health workforce investment will also make the task of achieving a more efficient and equitable distribution of staff much easier.

It is also important to recognize that health workforce, while indispensable to any strategy to strengthen health systems, is not a sufficient investment on its own. The workforce will not be effective if other elements of the health system are not also strengthened. This includes a need for broader systemic reform to strengthen financing and decision-making authority at sub-national levels to facilitate local training, recruitment, and support provided to both health workers and populations at those levels [14].

Overall, the case study illustrates that producing a sea-change in a country’s health system level is not just a matter of will or a large enough international investment that might emerge from a new understanding that the world’s health security is only as strong as its weakest health system which the Ebola crisis highlighted. Sustainability of substantially strengthened health systems needs to be achieved within the limited capacities of the poorest economies. The world’s health security is ultimately dependent on strategies that strengthen health systems alongside economic development (modeled in our analysis by growing health budgets over time), and that reduce global inequality in the process.

## Additional files

Additional file 1: Rural, urban, and total population projections for Liberia, Guinea and Sierra Leone: 2014-2030. This is the population data that has been used to calculate projected workforce densities and target densities. (DOCX 20 kb)

Additional file 2: Annual salary and training cost assumptions for Liberia, Sierra Leone, and Guinea. (DOCX 15 kb)

Additional file 3: Budget forecast, workforce cost and projections, 2014, 2020, 2030. (DOCX 18 kb)

## Abbreviations

CHW: Community health worker
EHRP: Emergency Human Resources Plan
HRH: Human resources for health
IMF: International Monetary Fund
UHC: Universal health coverage
WHO: World Health Organization

## Footnotes

1
https://www.nbcnews.com/storyline/ebola-virus-outbreak/runaway-doctors-missing-supplies-cripple-care-ebola-hit-liberia-n220686

2
http://www.imf.org/external/pubs/ft/weo/2016/01/weodata/index.aspx
