# Supplementary material for: Never again? Challenges in transforming the health workforce landscape in post-Ebola West Africa
Source: Hum Resour Health. 2019 Mar 7;17:19. doi: 10.1186/s12960-019-0351-y (PMC6407225; doi:10.1186/s12960-019-0351-y)
Supplement: Supplementary file 1 — Rural, urban, and total population projections for Liberia, Guinea and Sierra Leone: 2014-2030. This is the population data that has been used to calculate projected workforce densities and target densities. (DOCX 20 kb) [file 12960_2019_351_MOESM1_ESM.docx]

| Country |  | 2014 | 2015 | 2016 | 2017 | 2018 | 2019 | 2020 | 2021 | 2022 | 2023 | 2024 | 2025 | 2026 | 2027 | 2028 | 2029 | 2030 |
| --- | --- | --- | --- | --- | --- | --- | --- | --- | --- | --- | --- | --- | --- | --- | --- | --- | --- | --- |
| LIBERIA | Rural population growth (annual %) | 0.028 |  |  |  |  |  |  |  |  |  |  |  |  |  |  |  |  |
|  | Urban population growth (annual %) | 0.043 |  |  |  |  |  |  |  |  |  |  |  |  |  |  |  |  |
|  | Rural population | 2982571 | 3066742 | 3153288 | 3242276 | 3333776 | 3427857 | 3524594 | 3624061 | 3726335 | 3831495 | 3939623 | 4050802 | 4165119 | 4282662 | 4403522 | 4527793 | 4655571 |
|  | Urban population | 1413983 | 1474294 | 1537178 | 1602744 | 1671107 | 1742386 | 1816705 | 1894193 | 1974988 | 2059228 | 2147061 | 2238641 | 2334127 | 2433686 | 2537492 | 2645725 | 2758574 |
|  | Total | 4396554 | 4541036 | 4690466 | 4845020 | 5004883 | 5170243 | 5341299 | 5518255 | 5701323 | 5890723 | 6086684 | 6289443 | 6499246 | 6716348 | 6941014 | 7173518 | 7414145 |
|  |  |  |  |  |  |  |  |  |  |  |  |  |  |  |  |  |  |  |
| GUINEA | Rural population growth (annual %) | 0.021 |  |  |  |  |  |  |  |  |  |  |  |  |  |  |  |  |
|  | Urban population growth (annual %) | 0.040 |  |  |  |  |  |  |  |  |  |  |  |  |  |  |  |  |
|  | Rural population | 6924139 | 7067655 | 7214146 | 7363673 | 7516299 | 7672089 | 7831108 | 7993423 | 8159102 | 8328215 | 8500833 | 8677030 | 8856878 | 9040454 | 9227834 | 9419099 | 9614328 |
|  | Urban population | 3704835 | 3851988 | 4004985 | 4164060 | 4329452 | 4501414 | 4680207 | 4866100 | 5059377 | 5260331 | 5469267 | 5686501 | 5912364 | 6147197 | 6391359 | 6645218 | 6909160 |
|  | Total | 10628974 | 10919643 | 11219131 | 11527733 | 11845752 | 12173503 | 12511314 | 12859523 | 13218479 | 13588546 | 13970100 | 14363531 | 14769241 | 15187651 | 15619193 | 16064317 | 16523488 |
|  |  |  |  |  |  |  |  |  |  |  |  |  |  |  |  |  |  |  |
| SIERRA LEONE | Rural population growth (annual %) | 0.018 |  |  |  |  |  |  |  |  |  |  |  |  |  |  |  |  |
|  | Urban population growth (annual %) | 0.031 |  |  |  |  |  |  |  |  |  |  |  |  |  |  |  |  |
|  | Rural population | 5297699 | 5391072 | 5486091 | 5582785 | 5681183 | 5781316 | 5883213 | 5986906 | 6092427 | 6199808 | 6309081 | 6420281 | 6533440 | 6648594 | 6765777 | 6885026 | 7006376 |
|  | Urban population | 1139767 | 1174549 | 1210393 | 1247331 | 1285396 | 1324622 | 1365046 | 1406703 | 1449632 | 1493870 | 1539459 | 1586439 | 1634853 | 1684744 | 1736157 | 1789140 | 1843739 |
|  | Total | 6437465 | 6565621 | 6696484 | 6830116 | 6966579 | 7105938 | 7248259 | 7393610 | 7542059 | 7693678 | 7848540 | 8006720 | 8168293 | 8333337 | 8501934 | 8674165 | 8850115 |
|  |  |  |  |  |  |  |  |  |  |  |  |  |  |  |  |  |  |  |
|  | Population projections are based on 2014 population estimates and 2014 rural and urban annual growth rates | | | | | | | | | |  |  |  |  |  |  |  |  |
|  | Growth rate are calculated using World Bank population estimates and urban ratios from the United Nations World Urbanization Prospects. | | | | | | | | | | | |  |  |  |  |  |  |

**Title: Rural, Urban and Total Population projections for Liberia, Guinea and Sierra Leone: 2014-2030**

**Description: This is the population data that has been used to calculate projected workforce densities and target densities**
