# Supplementary material for: Never again? Challenges in transforming the health workforce landscape in post-Ebola West Africa
Source: Hum Resour Health. 2019 Mar 7;17:19. doi: 10.1186/s12960-019-0351-y (PMC6407225; doi:10.1186/s12960-019-0351-y)
Supplement: Supplementary file 2 — Annual salary and training cost assumptions for Liberia, Sierra Leone, and Guinea. (DOCX 15 kb) [file 12960_2019_351_MOESM2_ESM.docx]

|  | Annual Salary (USD) | Cost of training (USD) |
| --- | --- | --- |
| **LIBERIA** |  |  |
| Specialist medical practitioners | 5736 | 25000 |
| General medical practitioners | 5027 | 25000 |
| Registered Midwife | 2424 | 3000 |
| Midwifery professionals | 2144 | 3000 |
| Nurse professional | 2538 | 4500 |
| Registered Nurse | 2300 | 4500 |
| **GUINEA** |  |  |
| Specialist medical practitioners | 1317 | 2884 |
| General medical practitioners | 1160 | 2884 |
| Midwife | 1070 | 1647 |
| Nurse | 378 | 1440 |
| Registered Nurse | 1083 | 1440 |
| **Sierra Leone** |  |  |
| Specialist medical practitioners | 33783 | 100000 |
| General medical practitioners | 11754 | 100000 |
| Midwifery professionals | 3757 | 10000 |
| Nurse professional | 3859 | 20000 |

**Annual salary and training cost assumptions for Liberia, Sierra Leone and Guinea**

SOURCE: Annual salary estimates obtained from payroll data from Government Health Departments in Liberia, Sierra Leone and Guinea. Training cost estimates from the One Health Tool used by the Governments in Guinea and Liberia for Investment Cases and the HRH Strategic plan SL Dec 2012 for Sierra Leone.

NOTES: Average costs were calculated per health cadre (for example, average annual costs and training costs for doctors calculated using Specialists and General Practitioners)
