# Supplementary material for: Never again? Challenges in transforming the health workforce landscape in post-Ebola West Africa
Source: Hum Resour Health. 2019 Mar 7;17:19. doi: 10.1186/s12960-019-0351-y (PMC6407225; doi:10.1186/s12960-019-0351-y)
Supplement: Supplementary file 3 — Budget forecast, workforce cost and projections, 2014, 2020, 2030. (DOCX 18 kb) [file 12960_2019_351_MOESM3_ESM.docx]

|  | **Budget Forecast, Workforce Cost and Projections, 2014, 2020, 2030** | | | |  |  |  |  |
| --- | --- | --- | --- | --- | --- | --- | --- | --- |
|  | **Projection** | **Workforce cost ($US M)*** | **GDP ($US M)** | **Workforce Cost (% GDP)** | **Govt. Expenditure ($US M)** | **Workforce Cost as % Govt expend.** | **Govt. Health Expenditure ($US M)**** | **Wage Bill as % Health Expenditure** |
| **Guinea** | Current^a^ | 11.8 | 6699 | 0.18% | 1775.24 | 0.66% | 65.15 | 18% |
|  | Total workforce with scale-up (2020)* | 17.5 | 8765 | 0.20% | 2427.91 | 0.72% | 89.10 | 20% |
|  |  | 10.4 | 8765 | 0.12% | 2427.91 | 0.43% | 89.10 | 12% |
|  | Plan doctors, nurses and midwives, total cost 2020*** |  |  |  |  |  |  |  |
|  | 2.5/1000 population doctors, nurses and midwives total cost 2020*** | 15.9 | 8765 | 0.18% | 2427.91 | 0.65% | 89.10 | 18% |
|  | 2.5/1000 population doctors, nurses and midwives total cost 2029 (at 2020 national data projections)*** | 74.3 | 8765 | 0.85% | 2427.91 | 3.06% | 89.10 | 83% |
|  |  |  |  |  |  |  |  |  |
|  | (on basis of 5% growth rate 2020-2030)*** | 74.3 | 14277 | 0.52% | 3954.79 | 1.88% | 145.14 | 51% |
| **Liberia** | Current^a^ | 37 | 2012 | 1.84% | 645.85 | 5.73% | 80.1 | 46% |
|  | Total wage bill with scale-up (2020)* | 48.8 | 3011 | 1.62% | 909.32 | 5.37% | 112.76 | 43% |
|  |  | 44.9 | 3011 | 1.49% | 909.32 | 4.94% | 112.76 | 40% |
|  | Plan doctors, nurses and midwives, total cost 2020*** |  |  |  |  |  |  |  |
|  | 2.5/1000 population doctors, nurses and midwives total cost 2020*** | 27.2 | 3011 | 0.90% | 909.32 | 2.99% | 112.76 | 24% |
|  | 2.5/1000 population doctors, nurses and midwives total cost 2029 (at 2020 national data projections)*** | 69 | 3011 | 2.29% | 909.32 | 7.59% | 112.76 | 61% |
|  | (on basis of 5% growth rate 2020-2030)*** | 69 | 4905 | 1.41% | 1481.19 | 4.66% | 183.67 | 38% |
|  |  |  |  |  |  |  |  |  |

|  | NOTES:  *Costs for ‘Baseline Scenario’ used for all workforce costs – attrition 10%, drop-out rate 20%, employment rate 50%  ***Total cost estimates for doctors, nurses and midwives represent those at progress toward target 2020 and 2029. For 2.5/1000 population projection, target date used is 2030. |
| --- | --- |
|  | ^a^Current estimates are based on 2016 workforce cost projections (based on Baseline Scenario) compared to 2014 levels of GDP and government expenditure.  Government exp calculated using GDP current prices (US$ millions) and General Government Expenditure as % GDP for 2014 (Guinea 26.5% and Liberia 32.1%) and 2020 (Guinea 27.7% and Liberia 30.2%) Govt. health expenditure calculated using Govt. expenditure as a % GDP used 3.67% for Guinea, 12.4% for Liberia, and 7.1% for Sierra Leone .  SOURCE for GDP and Govt. expenditure data: International Monetary Fund, World economic Outlook Database, April 20162020 estimates are calculated using target percentages discussed in government fiscal space publications. |
|  |  |
|  |  |
